# Behind every good research there are data. What are they and their importance to forensic science

**DOI:** 10.1016/j.fsisyn.2024.100456

**Published:** 2024-02-08

**Authors:** Lucina Hackman, Pauline Mack, Hervé Ménard

**Affiliations:** Leverhulme Research Centre for Forensic Science, University of Dundee, Nethergate, Dundee, DD1 4HN, UK

## Abstract

Data underpinning science have become one of the most precious assets in research, and while the principles of FAIR (Findable, Accessible, Interoperable and Reusable) have been put forward as a guide to how to approach data handling, data sharing and long-term storage still remain a challenge for many research areas including forensic science. The reporting and the sharing of data can be made easier by giving them structure, the use of suitable labels and the inclusion of descriptors collated into metadata prior to their deposition in repositories with persistent identifiers. Such a systematic approach would strengthen the quality and the integrity of research while providing greater transparency to published materials.

## 1 Introduction

Data can be defined as facts (small, large, seemingly simple or random) that can be stored and interrogated. Often used interchangeably with the term information, there is a subtle difference between the two. The term data refers to the raw details from which information is subsequently derived. What is of importance is the availability of generated data to the wider community, especially those involved in analysis and evaluation, such as the forensic science community.

Publishing is an established process aimed at increasing knowledge whilst creating permanent and searchable records. It is expected that the content of published material is referenced in any newly created output, but in recent years the data underpinning the science are also often cited. Citing the underpinning data relies on the data being first made available for access and repurposing, something that, whilst often elusive, is becoming increasingly important. Numerous publications have reported on data availability, its importance and benefits to the community and how this can be leveraged. To ensure that the data depended upon in any given publication, can be used and be useful, the principles of FAIR (Findable, Accessible, Interoperable and Reusable) have been put forward as a guide to how to approach data handling [[1], [2], [3]]. FAIR principles are increasingly used by publishers, editors and funders to outline research data preservation. However, adherence to FAIR principles or even the simple concept of data availability remains a challenge, particularly for research areas or disciplines that have yet to implement their own solutions. One of these areas is forensic science, which is not one single science type but is multi-disciplinary in nature, requiring as a result, adapted and adaptable solutions depending on the reported topic of interest.

This article is intended to provide some basic descriptions of some of the concepts already reported in the literature, such as FAIR, to the forensic science community who may find relevant to their work. The aim of this article is to specifically focus on what data are since they are the foundation on which all else must stand. This is done from a general scientific perspective which will find relevance in a broad range of disciplines and areas of research including forensic science. The topic is further extended toward the principle of a data management plan, as a possible approach that allows those undertaking scientific research to assist forensic science researchers and practitioners who are dealing with data. This informs how data can be managed from the early onset of a project as part of the workflow. The paper then discusses how data can be visualised and presented for use by forensic scientists by using examples of existing datasets.

## 2 Background: what are data?

Whilst definitions of data appear clear, referring to facts, numbers, or observations, it is also evident that when it comes to data content, there is no homogeneity, causing easy confusion in relation to the ways in which data are handled, interpreted and presented. Clearly describing the differences between data types and fully understanding data classification permits the correct use of measurements, their analysis and their interpretation, but also allows us to define adequate solutions for the storage and presentation of data for others to access and use.

**Fig. 1:**
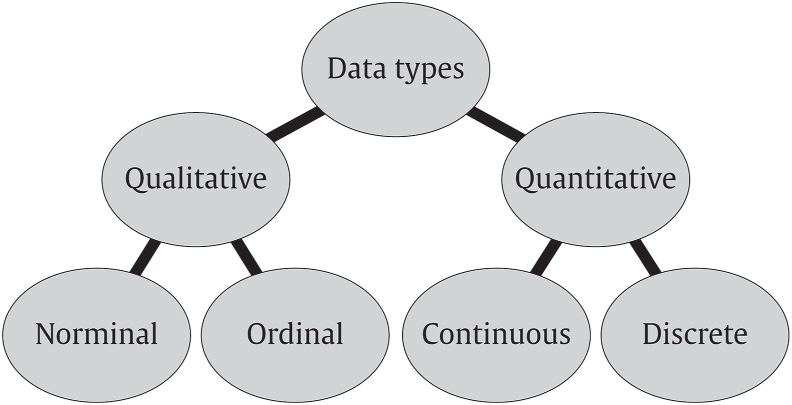
Data types and general terms.

At the highest level, data exist as either quantitative or qualitative (Fig. 1). Quantitative data refer to numbers and measurements objectively collected while qualitative data include details and descriptors that are not easily measured but can still be reported subjectively.

Further distinctions can be made within these two general classifications (Fig. 1). Discrete data refers to values that are distinct and separate, counted and not measured. Discrete data is presented as integers and cannot be subdivided into smaller parts. Continuous data refers to values that can be measured but not counted. Continuous data can be divided further into ratio and interval variables.

Interval variables are responses found on an ordered scale with defined spacing, for which the difference (subtraction or addition) between two numbers is meaningful but not their multiplication or division.

Qualitative data represent characteristics that are generally, but not always, non-numerical values. Nominal data consists of discrete units that describe general attributes, such as a person's gender, location, language, *etc*. Nominal data are called dichotomous when the choice is between just two possible outcomes.; Ordinal data are similar to nominal data but also provide an order of scale. This data “classification” has roots dating back as far as the first half of the 20th century when social scientists; especially in psychology, sought to provide measurements to their studies in a similar way to that seen in physics or chemistry. The difficulty these more qualitatively based fields of research experienced during this process started the conversation toward a possible expansion of the definition of “measurement”, but as can be imagined, this met with objections. Nonetheless, Stevens [4] proposed a new definition of “measurement” based on rules with different levels or scales which categorise measurement as being nominal, ordinal, interval, and ratio. The assignment to the appropriate scale of measurement given by Stevens is based on the relationship between data and their mathematical properties. The framework established by Stevens is still in use, but it is also subject to debate for the controversies it brings. Stevens' classification has an emphasis on a mathematical representation of data, but in doing so it comes with the flaw that the absence of measurements could potentially lead to data exclusion. Based on this argument, social scientists noticing the absence of measure between their collected details, or simply the absence of numbers, can incorrectly assume their seemingly unrelated facts do not classify as data. Turning the argument in the opposite direction and looking back at the definition for data, all collected facts should be considered as data (and this regardless of their mutual relationship) while treating established measurements as information on which conclusions can be built.

Data are probably one of the most valuable resources a business can have since at a very basic level they can be monetised. Data are more than this though and can also help demonstrate success, guide strategic decisions, inform on growth potential, improve processes or solve problems *etc*. However, for data to be of value, they need to be classified so that the appropriate operation can be performed to allow them to be properly understood and displayed. This display of data is known as data visualisation and can prove to be invaluable for those utilising it.

### 2.1 Reporting data

Data will be different depending on its origin but on their own it is straightforward to report. This may be, for example, a photographic image of a specimen or a scene. It is commonly the case that multiple facts or details need reporting at the same time, and these combine to form a dataset. A dataset is a structured collection of data that have been compiled and presented. Taking this concept to the next level, multiple datasets can be organised to form databases. The challenge here is not simply to report data but, to give structure to a collection of data, in order that it can be searchable and presentable for others to access. However, importantly, the idea that the place for a dataset is in the supplementary information or that the task of compiling data is no more than just putting all the files into a folder prior to sharing, is erroneous and results in poorly presented data that become difficult to understand and parse. To the first misconception and returning to the definition of data previously given, information is built on data and the supplementary information is a document, where additional information supporting the content of the main text should be found [5]. To the second point, while it is a positive move, a simple list of files that may come in multiple types (i.e., text, image, sound or video) without context, description or metadata, does not aid the reader nor facilitate queries, if accessed using a computer programme. Unfortunately, when it comes to structuring data into a dataset, there is no all-in-one solution but only some guidelines that can be followed.

As a first essential requisite, structuring data into a dataset should not lead to the loss of details. This means that all essential facts should be provided alongside the data. One example is the responses of a survey by Anagnostou et al. [6], dataset available at https://doi.org/10.1371/journal.pone.0121409.s002a and an extract presented in Table 1. This dataset by Anagnostou et al. [6] is one example among many that could have been chosen from the published literature. It was selected for its simplicity in presenting data, and it does not reflect the scientific findings of the authors’ work. The dataset provides the answers given by the participants but in doing so prevents details on the possible choices given in the survey. From the questions “Compliance *with* …“, “*Expectation to* …” and “*Awareness off* …“, it seems the participants had a choice between four answers: “*Very important*”, “*Important*”, “*Not very important”* and “*Not important at all*”; a typical selection that would be given for a Likert scale survey. It would be reasonable to ask if, just looking at the dataset, other answers were also available but not selected by any of the participants. This cannot be answered directly by simply reading the dataset although in their article, Anagnostou et al. [6] specified that the respondents could answer these questions in one of four-ways. However, no detail is given regarding the questions about the experience of the respondent (i.e., “*How long* …” and “*How long* … *population*?“). The consistency in the responses seems to indicate that a choice was offered, but with just “*More than 5 years*” and “*Between 2 and 5 years*” as well as “*I only have experience in ancient DNA studies*” being given in the dataset, it is impossible to establish what were, if any, the other alternatives.

**Table 1:** Extract of dataset reported by Anagnostou et al. [6] and available at: https://doi.org/10.1371/journal.pone.0121409.s002. Selected rows and columns to include all possible responses given by the participants.

		Focusing on …					…
Ids	How long …	Compliance with …	Expectation to …	Awareness of …	Awareness that …	How long … populations?	…
ID1	More than 5 years	Important	Very important	Very important	Very important	More than 5 years	…
ID2	More than 5 years	Very important	Not very important	Very important	Very important	More than 5 years	…
…	…	…	…	…	…	…	…
ID6	More than5 years	Very important	Not very important	Important	Important	I only have experience in ancient DNA studies	…
…	…	…	…	…	…	…	…
ID18	From 2 to 5 years	Important	Not important at all	Very important	Very important	From 2 to 5 years	…
…	…	…	…	…	…	…	…

It may be tempting to create streamlined datasets or present aggregated results that might appear clear to the readers but that is not the purpose of datasets. Regardless of how complicated datasets may look, the data they contain should be comprehensive. While not ideal in the case of the study by Anagnostou et al. [6], the authors should still be recommended for making the individual responses available. There are many datasets available in the literature and an example of a complete dataset (accessible using the statistical software IBM SPSS Statistics) is available in the study by Tenopir et al. [7], which is an illustration of the second point of this article about exposure and learning how to do it effectively.

Structuring data into a dataset is not something traditionally taught, but it is something that can be learned from viewing and understanding datasets already made available alongside the literature in many journals or websites (DRYAD, Harvard Dataverse, Open Science Framework, Zenodo, UK data archive, institutional repositories, *etc*.). At its core, publishing data is a public disclosure that aims to make the research traceable and reusable for future work. Using this argument, it would be perfectly reasonable to build new datasets with structures similar to one already published, thus facilitating an efficient and direct comparison between studies and other reference materials. Tenopir et al. [7], for example, give a good illustration of how to present a dataset and survey responses and any new studies could follow a similar route in structuring their datasets without the need to start from the basics. Unfortunately, it may not always be possible to discover a reference dataset that will provide the perfect template, and, in these cases, more background work is required to give structure to the data, even though other datasets available in non-related research, can still be inspirational when completing this task. Lastly, some unfamiliarity with dataset file formats or restrictions in accessing software would likely be an impeding factor for sharing and long-term data curation.

The pace of change in digital technology presents a challenge for the long-term preservation of data, an issue for anyone including those who own the data. To avoid losses, reasonable actions are required to help preserve data content and functionality while maintaining its value and use for future research. However, while often being a recurring topic of conversation between data scientists or librarians and researchers in academic institutions, particularly at the time of submission of a document for publication, there is a paucity of literature on data curation. Many academic institutions and businesses, have implemented measures or proposed guidelines to lessen this data curation challenge and gaining some familiarity with these instructions when available, is recommended. One approach to ensuring that file contents remain useable over time is to utilise file formats with the highest probability of long-term preservation (Table 2).

**Table 2:** Table of recommended formats for different data content types. Adapted from Georgia Southern University [8] libraries guides.

Content types	Probability for long-term preservation		
	High	Medium	Low
Text	• Plain text [encoding: USASCII, UTF-8, UTF-16 with BOM] (*.txt)• XML – includes XSD/XSL/XHTML, *etc*.• PDF/A-1 [ISO 19005 1] (*.pdf)	• Cascading Style Sheets (*.css) • DTD (*.dtd) • Plain text [ISO 8859 1 encoding] • PDF – embedded fonts (*.pdf) • Rich Text Format 1.x (*.rtf) • HTML – include a DOCTYPE declaration • SGML (*.sgml) • Open Office (*.sxw/*.odt)• OOXML [ISO/IEC DIS 29500] (*.docx) • markdown (.md) or R markdown (.Rmd)	• PDF (*.pdf) (encrypted) • Microsoft Word (*.doc) • WordPerfect (*.wpd) • DVI (*.dvi) • All other text formats
Raster Image	• TIFF version 6 uncompressed (.tif) • JPEG2000 (lossless) (*.jp2) • PNG (*.png)	• BMP (*.bmp)• JPEG/JFIF (*.jpg) • JPEG2000 – lossy (*.jp2) • TIFF – compressed • GIF (*.gif) • Digital Negative DNG (*.dng)	• MrSID (*.sid) • TIFF (in Planar format) • FlashPix (*.fpx) • PhotoShop (*.psd) • RAW • JPEG 2000 Part 2 (*.jpf, *.jpx) • All other raster image formats
Vector Graphics	• SVG – no Java script binding (*.svg)	• Computer Graphic Metafile [CGM, WebCGM] (*.cgm)	• Encapsulated Postscript (EPS) • Macromedia Flash (*.swf) • All other vector image formats
Geospatial	• ESRI Shapefile (*.shp, *.shx, *.dbf; optional - *.prj, *.sbx, *.sbn) • geo-referenced TIFF (*.tif, *.tfw) • CAD data (*.dwg) • tabular GIS attribute data • Keyhole Mark-up Language (KML) (*.kml)	• ESRI Geodatabase format (*.mdb) • MapInfo Interchange Format (*.mif) for vector data	
Audio	• AIFF [96 kHz 16bit PCM] (*.aif, *.aiff) • WAV [96 kHz 24bit PCM] (*.wav)	• SUN Audio (uncompressed) (*.au) • Standard MIDI (*.mid, *.midi) • Ogg Vorbis (*.ogg) • Free Lossless Audio Codec (*.flac) • Advance Audio Coding (*.mp4, *.m4a, *.aac) • MP3 (MPEG-1/2, Layer 3) (*.mp3) • Audio Interchange File Format (AIFF) (.aif)	• AIFC (compressed) (*.aifc) • NeXT SND (*.snd) • RealNetworks ‘Real Audio’ (*.ra, *.rm, *.ram) • Windows Media Audio (*.wma) • Protected AAC (*.m4p) • WAV (compressed) (*.wav) • All other audio formats
Video	• Motion JPEG 2000 [ISO/IEC 15444 4)??] (*.mj2) • AVI (uncompressed, motion JPEG) (*.avi) • QuickTime Movie (uncompressed, motion JPEG) (*.mov)	• Ogg Theora (*.ogg) • MPEG-1, MPEG-2 (*.mpg, *.mpeg, wrapped in AVI, MOV) • MPEG-4 (H.263, H.264) (*.mp4, wrapped in AVI, MOV)	• AVI (others) (*.avi) • QuickTime Movie (others) (*.mov) • RealNetworks ‘Real Video’ (*.rv) • Windows Media Video (*.wmv) • All other video formats
Spreadsheet/Database	• Comma Separated Values – ASCII or Unicode (*.csv) • Delimited Text – ASCII or Unicode (*.txt) • SQL DDL • Structured text or mark-up file containing metadata information (DDI XML, JSON *etc*.) • tab-delimited file (.tab) including delimited text of given character set with SQL data definition statements where appropriate	• dBase (*.dbf) • OpenOffice (*.sxc/*.ods)• OOXML (ISO/IEC DIS 29500) (*.xlsx) • Excel 2007 or newer (*.xlsx) • MS Access (.mdb/.accdb) delimited text of given character set – delimiter characters not used in data (*.txt)	• Excel – 2003 or older (*.xls) • All other spreadsheet/database formats
Virtual Reality	• X3D (*.x3d)	• VRML (*.wrl, *.vrml) • U3D (Universal 3D file format)	• All other virtual reality formats
Computer Programs	• Uncompiled source code, (*.c, *.c++, *.java, *.js, *.jsp, *.php, *.pl, *etc*.)		• Compiled /Executable files (EXE, *.class, COM, DLL, BIN, DRV, OVL, SYS, PIF)
Presentation		• OpenOffice (*.sxi/*.odp) • PowerPoint 2007 or newer (*.pptx)• OOXML (ISO/IEC DIS 29500) (*.pptx)	• PowerPoint 2003 or older (*.ppt) • All other presentation formats
Instrumentation^a^	• NMR, IR, Raman, UV, Mass Spectrometry converted to JCAMP (*.jdx) • GC/MS converted to mzML, mz5 or ms1 with structured text for metadata • XPS: vamas (.*vms) • XRD: ASCII (*.dat)		• Original files with structure text containing metadata information

a Whenever possible, instrument data should be converted to high probability for long term preservation file formats.

The complex appearance of content inside Table 2 is simply because there is no one-fits-all solution when it comes to long term data preservation. Each individual research dataset will have its own needs depending of the type of study that is being carried out and the type of data that has been collected. It is recommended that scholars make their own list of file types based on the work they do. The important point of Table 2 is to draw attention to the need to check the data export format if long-term preservation is sought. This is essential if data are intended to be shared or if there is a risk of technological obsolescence, which realistically is nearly always the case. Converting files to formats that may be more suitable for long term preservation can lead to the loss of details, some of which might be potentially relevant to the project. This compromise between data conversion and the retention of all appropriate facts needs to be assessed from the onset of the work. Projects and studies can accumulate a variety of data, with various content types and their grouping by content with the addition, of metadata and data description, will result in the desired structured collection.

Metadata are data that in turn, give further information about the data, so essentially they are data about the data. Metadata are the foundation of digital data curation, making the data findable, accessible, interoperable and reusable, following the FAIR principles [[1], [2], [3]]. Metadata usually contain a set of structured details aimed at facilitating access management and content while also describing the resource, their location and how they can be retrieved by users. The various elements that make the metadata can be categorised by the functions they support. These defined functions are usually standardised with controlled vocabularies and name authorities to guide the content of the metadata. When grouped together and designed for a specific purpose, these are called metadata schemas. Many schemas are used as standards across data repositories and disciplines, and the schemas developed and maintained by organisations or institutions are called metadata standards. Several of these standards can be found at Digital Curation Centre [9] or the Research Data Alliance Metadata Standards Directory [10] with additional information provided in registers like FAIRsharing.org or re3data.org.

By their contents, the metadata standards are intended to be machine readable and usually come with an XML (eXtensible Markup Language) file format. However, metadata are not something that is normally put together by the author(s) of the data, or at least not directly. The details entered by the user, during the submission to a repository, are used to create the metadata that will be attached to the dataset. In other words, the information entered during submission are used to present a summary or the highlights of the dataset, or to return matching results following a query using the search box. For that reason, the visibility of a dataset will only be as good as the information entered in the first place. There are many metadata schemas and standards available to the community all having mandatory or optional fields to complete. For being the common denominator across the majority of datasets, metadata are predominantly focused on the bibliometry (required elements for authors, affiliation, licence, copyright, *etc*.) instead of the voluntary inclusion of a detailed description of the data. In this case, while meeting the FAIR principles, it can fail on data reusability since the lack of context or explanation may prevent external users from fully understanding the content of the dataset.

To address the issue of context, an additional file can be included with the data. This could be for example the inclusion of a Readme file. A Readme file is a text (.txt) document used to introduce a project and what is being uploaded. Readme files are often provided with software and codes to provide instructions regarding installation and minimum system requirements. As plain text, there is no limit to their content length which should be complementary and explanatory of the files they are associated with. It is however important to indicate that the Readme file should remain general instead of being a detailed description of all the data. For example, in the case of a dataset containing images, videos, audio or any of the outputs collected with an electronic device or instruments, the Readme file will be aiming at the generalities applicable to all or groups of files (e.g., type of instruments, collection date range, the general location like country, region, city, *etc*., calibration details, *etc*.). All matters specific to individual files should be placed in a separate document (.txt, .csv or equivalent) which will list all the files with any additional facts and details that could not be captured within the data but judged important for future interpretation. A description of all the columns should however be included in the Readme file. The Readme file may also include technicalities on the applied file conversation step (to increase the probability of long-term preservation) as well as the details of content loss.

It is important to understand that there is no one size fits all solution when it comes to creating structured data due to the infinite diversity of research and data they generate. As a result, some form of adaptation and flexibility to the general template, previously described, will always be required to complete this task. The responsibility to carry out this work lies with the researchers simply submitting the minimum details will not facilitate the understanding of the data, making the derived findings less reproducible as well as impacting the future studies, that may have benefited from or could have been built on, this initial research. Leaving this task to the end is likely to be an inefficient process especially for projects with large datasets or a variety of file types. Time pressure between publication and data submission can be detrimental to completing the task to ensure that the data are optimally presented. The decision to structure the data needs to be carried out from the conception of work, even prior to data collection, as part of a comprehensive research strategy, so that it becomes a direct part of the methodology and dealt with gradually during the duration of the study or project.

### 2.2 Data management plan

A data management plan is a written document that explains how the gathered data will be managed from the time of collection to their final storage in an organised dataset. Many academic institutions provide detailed descriptions and examples of data management plans with additional strategies and discussions reported in several publications; see for example [[7], [11], [12]]. Gebru et al. [13] take the data management plan a step further and advocate the inclusion of an accompanying datasheet, that details the composition, collection process, preprocessing/cleaning/labelling, uses, distribution and maintenance plan associated with each dataset. They outline questions that should be answered within this comprehensive datasheet, which allows any future users of the data to understand not only how they are stored and managed but also their origins. With so much information already at hand, the purpose here is not to look at what is a data management plan but why it is important as part of a study.

Putting together a research project, applying for a government grant or completing a project description for an industrial development program, often requires a data management plan. The writing of the data management plan can be perceived as being an excessive administrative task which only requires the minimum attention, but equally it is important to remember that the data that are collected is a valuable asset. With this in mind, the data management plan should address resource allocation and by process estimate any costs that might be incurred. Regardless of the amount of data being collected, data storage require resources which come at a cost. The deposition of data into repositories, the support of that repository for the duration of the project and beyond, need to be fully evaluated in order to be adequately financed. The selection of a repository in which the data will be deposited should be made in accordance of the guidelines set by the author(s)’ institution. This will be for example, the cost per gigabyte, or terabyte of server or cloud space, as well as the infrastructure surrounding this. One motivation of the data management plan is to estimate from the onset of the project, the type and volume of data that will be collected, thus allowing an early decision about how this can be transformed into a structured set of data, with a high probability for long term organisation and storage. In this way, the data management plan becomes an essential tool for describing the various steps involved in creating the structured data and a reference guide to the authors, turning a potentially tedious task into an efficient process.

### 2.3 Application to forensic science

There is a need for data in forensic science to support and provide the reliability, viability, and conclusions of forensic examinations. Following the damaging assessments given in the National Research Council [14] and 2016 President's Council of Advisors on Science and Technology [15] reports, calls have been made to encourage the publication of datasets, many of which are related to digital evidence, but also in other areas such as pathology [16], fibres [17] and shoemarks [18]. Whilst the principles of FAIR have been well described and explained and are starting to be implemented in research and in relation to some research fields, the types of data and the timeliness of the data required to underpin sound forensic science present a number of challenges to these that have yet to be taken into consideration at all levels. Some of these challenges are financial, for example the cost of a data repository and its maintenance can be expensive. Other challenges are rooted in privacy and security concerns and others are centred on the data types themselves since some data are related to personal identification of individuals, such as DNA. A point to consider is the usefulness and the timeliness of data which means that data should be of quality and not become obsolete before they are available to use, given the time that it takes for research to be undertaken and published. Data obsolescence and quality are separate aspect that can still have connection; data of poor quality are more likely to become obsolete. Good data quality should be sought either in their creation or usage and this can be evaluated by analysing various attributes or dimensions such as completeness, consistency, uniqueness, integrity, validity and accuracy [19,20]. There may however be limitations that inadvertently lead to a negative assessment of the data robustness as may occur in academic studies or research built from casework, because of sensitivity or confidentially issues, which means that there may not be a process by which the data can be verified for accuracy and validity [[21], [22], [23]].

Data and datasets should be welcomed as they are intended to promote research integrity and transparency. However, it is also equally important to remember that data submission alongside a publication or in a repository, is not an automatic guarantee of research excellence or its usefulness for future work. It is also not a guarantee that the data contained within the database would be useful to the forensic scientist whose work is based in data generated from a crime scene and whose aim and requirements are divergent from that of those undertaking scientific research [24,25].

In their work on the development of a footwear impression database for teaching and research purposes, Lin et al. [26] reported on an open-source dataset comprising of 214 high-quality impressions and 383 dust and blood impressions, (data available at http://4n6chemometrics.com/Downloads/WVU2019/). In the dataset, some of the images received enhancement and the generated outputs were submitted alongside the original ones. For the enhanced images, the letter E was added to the filename and the file naming protocol described in the manuscript. Full details regarding the applied image correction as well as other descriptors were given by Lin et al. [26] in the metadata although the authors decided to place both the original image name and its respective enhanced version (if available) on the same row. Such an approach may be intended to simplify content to make it more readable by its user, but it raises some difficult issues when attempting to directly machine-match the list of files to the metadata. It is also interesting to see that while the data collection by Lin et al. [26] was intended to expand upon the content of public datasets, the authors decided to produce their own structure instead of following the file naming convention and metadata descriptors already given in existing databases, an example being the one given by the Centre for Statistics and Applications in Forensic Evidence (data available at https://data.csafe.iastate.edu/DataPortal/#). It is perfectly acceptable that data collected in different studies may not cover the same parameters but some commonalities between the work should be sought, ideally using the same experimental benchmark, to validate the methodology and data acquisition protocols. In their study, Lin et al. [26], acknowledged some of the limitations of their work, for example having to rely on personnel that may have a different shoe size to the one used for the impression, or that slightly different dimensions may occur between the various scanning methods. The extent of sizing variation may be evaluated with image comparison; however, it is not possible to identify which impressions were generated by someone normally wearing a different shoe size and the wearer's size could have been recorded within the data description, as seen in the CSAFE dataset. Finally, a list of proprietary and open-source databases is provided by Lin et al. [26] and even though their research was published in late 2021, the 4 cited crime scene open-source datasets are hosted directly on websites, instead of using a link with a persistent identifier, such as a digital object identifier (DOI), and two of these datasets are not retrievable due to faulty links (i.e. webpages no longer accessible).

The issue of data persistence affects datasets and databases differently depending on whether the works are primarily academic or commercial in nature. Research carried out in an academic environment usually has very specific aims which are described in the initial project proposal. The collected data are expected to provide answers to a set of questions and while the data content can be very comprehensive, they remain focused on the intended study. These data can be described as one-off data because they are usually just attached to specific outputs, and they receive no future development after the project completion. This is generally the case for funded research that cannot secure continuation grants to allow further data collection and the maintenance of webpages and servers. On the other hand, for proprietary databases, subscription costs or the fixed charges to access and download are part of the business model to ensure the perennity of the provided services. As well as providing regular content updates to encourage subscribers to return, some of these databases may also permit their user base to upload their own data that can then be shared with the rest of the community. A well-known example is the Cambridge Crystallographic Data Centre (CCDC) which hosts over 1.1 million curated crystal structures collected by more than 460,000 distinct authors and from over 560,000 papers (as of Aug 2022). The CCDC database also contains 377 crystal structures related to explosives and 5945 to drugs, although not all may be relevant to forensic science since there is no structure classification falling under forensic. Another example of a database that relies on user contributions is the NIST Ballistics Toolmark Research Database (NBTRD). NBTRD is an open-access database of bullets and cartridge cases. User registration is only required to upload data but it is not necessary for search and download. There are currently 46 studies listed in the databases, covering a total of 1528 firearms, 4008 cartridges, 2937 bullets (mostly from the Los Angeles Police Department) and 23,859 images.

Databases built on the model of user contribution, as seen with the CCDC, the NBTRD and many others, offer systematic harmonisation between submitted datasets. As part of the data upload, specific fields and entries are required while some others remain optional. This implies data must already be in the expected format alongside conforming metadata. In doing so, submitting to a database offers longevity to the data for it remains easily accessible long after the end of the initial work. Furthermore, instead of having a simple link to a repository, data deposition in a database increases research visibility with the release of database updates.

While possessing numerous benefits, other factors merit consideration. Research impact is crucial not only for researchers, but also for funders seeking proof of return on their investment [27]. However, a study by Jensen et al. [28] which analysed impact statements submitted during the UK Research Excellence Framework of 2014 indicated that data alone only occasionally generated impact. Several methods have been proposed for analysing impact statements (see for example [29]. However it would be reasonable to state without much controversy that poor data visibility would be an impeding factor to explaining the significance of the research. Databases could be part of a solution to increase the visibility and reach of individual contributions.

In forensic science, many physical trace evidence (e.g. soil [30,31], pollen [32] benefit from having data disseminated in a standardised format. An example is drug evidence and especially new psychoactive substances (NPS) which have received significant research interest due to their increasing numbers and associated caseworks. Mardal et al. [21] and more recently Von Cüpper et al. [33] reported on the importance of combining lab-based observations with an online mass spectral database (HighResNPS) to obtain complementary information. Data stored in HighResNPS are kept up to date thanks to the contribution of many forensic laboratories around the world, creating a crowd-sourcing approach to ensure that the data deposited are not only consistent in nature and therefore accessible, but remain timely, ensuring they are available whilst currently applicable. While the content of HighResNPS currently only lists detected NPS found in drug seizures, new research by Skinnider et al. [34] has been built on this collective database to help with the early identification of new compounds that are not yet included in any reference lists.

Whilst there are many benefits to data sharing, there are also challenges and limitations that need to be considered, especially when related to data sharing in the forensic science domain. This is particularly the case for evidence such as DNA or fingerprints, but also any other sensitive personal data or security sensitive details [35]. For example, several studies have looked and discussed at the benefits of DNA databases [36,37]. Scientific advancement can be accelerated with greater inclusion and accessibility of data into databases, but not if it comes at the risk of being misused, resulting in restrictions and legislation that surrounds their use, maintenance and access. The idea of sharing too much data is also one to consider, since there may be security considerations that should be taken into account when sharing information with those who may misuse it in the future [38]. The provenance of data contained within datasets can mean that the data are not reusable for reference purposes of forensic science. Datasets must be comprised of data that are balanced and unbiased, this has proven to not be the case in population databases for instance and whilst not an insurmountable issue, remains one that occurs and reoccurs often due to the different aims inherent within research data and those required for forensic science purposes [22,39]

Forensic scientists (academics and practitioners) should draw inspiration from other research when making their data available. Utilising the work done by others can guide and be adjusted to meet new requirements. This inspiration could come from related research (on the same topic or area of interest), from research on entirely different subjects or disciplines – preferred if they best fit the actual requirements – or a combination of both. Such an approach could save time in organising the data in a fixed format (e.g., tables) to make them easily searchable and analysable. It can also facilitate in-depth comparisons between works. To achieve this goal, a proactive approach is required to address what may be perceived as a tedious task in handling and managing data. Many institutions have implemented measures or offer guidelines to alleviate this data curation challenge. Therefore, it is recommended to discuss the data needs with colleagues (e.g., forensic scientists, data engineers) in the early stages of a project in an attempt to minimise effort while ensuring that the data remain accessible, integral, and useable.

Independent of content, data deposition in a database does not guarantee its long-term preservation. Many publishers and journals now require data to be deposited in a repository prior to or as part of the submission process. As previously described, this can be accomplished by depositing data in DRYAD, Harvard Dataverse, institutional repositories, Open Science Framework, Zenodo, and so on, but doing so would often require researchers, who want to share their data in a dedicated database, to double their effort because what is required for both (i.e., file and data structure requirements in the repository and the database) may differ. One recommendation would be that a given database allows the uploader to pull the data of interest using any existing persistent identifier (e.g. DOI), linking it to for example, published work, while also allowing missing fields to be completed.

The inclusion of a persistent identifier such as a DOI would certainly make it easy for humans and machines to find the data, hence starting to satisfy the first FAIR principle. However, this is only one point in many and only a clear understanding of what data are in the first place can start to address the problem of data with insufficient content. The use of mixed methods in forensic science adds to the complexity, making this possibly its greatest challenge. A solution to consider would be to intentionally subset the data into fundamental blocks that can easily be managed. This decision could be made based on selected methodology, instrumentation, *etc*. The relationships between the various data would be demonstrated by using appropriate sample names (i.e., the use of the same name for samples appearing between sets of data) and in the findings presented in published work (linked to the data). Such a strategy would help in applying FAIR principles to forensic science, and it will also address the issue of data sensitivity for the studies that may decide to systematically make a no-data statement when in fact partially dealing with data that cannot be disclosed.

The multidisciplinary nature of forensic science creates a complex landscape for sharing data, this is further complicated by the origins of the data that are utilised to form the databases as well as legislative and accessibility issues. Data that are created in academic research environments should not only be curated for access by forensic scientists but, should also be relevant to the analyses that they need to undertake and be in a form that is useable by them. It is vital therefore that projects where these data are created are devised in collaboration with forensic scientists, ensuring that they can provide input into the data planning stage of the research.

Databases can prove vital to ensure that those undertaking forensic analyses are able to keep up-to-date and stay relevant, especially in fields that are rapidly changing, such as drugs. This needs to remain current and to curate data that are meaningful to a forensic analysis and are therefore central to the success of these databases. This can become a challenge where data are produced across legislative or country boundaries, potentially resulting in variations in the data recorded or impeding access to those seeking the data. Additionally, due to the multidisciplinary nature of forensic science, databases of varying types are scattered across the globe rather than the data required being located in one central repository that is accessible. There is an argument therefore for the maintenance of central databases to support each of the forensic sciences, from toxicology through to fibres to which access can be granted.

## 3 Conclusion

Data are the foundation on which everything else is built. A clear understanding of what data are is necessary to ensure their meaningful collection and recording. Careful consideration should be taken ahead of starting any project and the inclusion of a project management plan can help with that task. Such action can make it easier to report and share and thus give the opportunity to adhere to the principles of FAIR (Findable, Accessible, Interoperable and Reusable). This is applicable to all disciplines and research areas including forensic science. The multidisciplinary nature of forensic science creates its own challenges due to the frequent use of mixed methods, but a simple solution could be at hand when considering data in specific blocks instead of as a whole. That is why a clear understanding of what data are becomes critical. With this knowledge, the forensic community can come together to decide how data should be organised and shared to strengthen the quality and integrity of research while providing greater transparency to published materials.

## Data accessibility

No data supporting this work were generated.

## Funding Information

This research was funded by the Leverhulme Trust RC-1015-011.

## CRediT authorship contribution statement

**Lucina Hackman:** Writing – review & editing, Writing – original draft, Conceptualization. **Pauline Mack:** Writing – review & editing, Writing – original draft, Conceptualization. **Hervé Ménard:** Writing – review & editing, Writing – original draft, Conceptualization.

## Declaration of competing interest

None.
